# Assessment of Some Inflammatory Biomarkers as Predictors of Outcome of Acute Respiratory Failure on Top of Chronic Obstructive Pulmonary Disease and Evaluation of the Role of Bacteria

**DOI:** 10.5402/2012/240841

**Published:** 2012-06-21

**Authors:** Hanaa Ahmed Shafiek, Nashwa Hassan Abd-Elwahab, Manal Mohammad Baddour, Mohamed Mabrouk El-Hoffy, Akram Abd-Elmoneim Degady, Yehia Mohamed Khalil

**Affiliations:** Faculty of Medicine, Alexandria University, El-Kartoum Square, Alexandria 21526, Egypt

## Abstract

*Objective*. To study the value of the inflammatory markers (interleukin-6 (IL-6), interleukin-8 (IL-8), and C-reactive protein (CRP)) in predicting the outcome of noninvasive ventilation (NIV) in the management of acute respiratory failure (ARF) on top of chronic obstructive pulmonary disease (COPD) and the role of bacteria in the systemic inflammation. *Methods*. Thirty three patients were subjected to standard treatment plus NIV, and accordingly, they were classified into responders and nonresponders. Serum samples were collected for IL-6, IL-8, and CRP analysis. Sputum samples were taken for microbiological evaluation. *Results*. A wide spectrum of bacteria was revealed; Gram-negative and atypical bacteria were the most common (31% and 28% resp.; single or copathogen). IL-8 and dyspnea grade was significantly higher in the non-responder group (*P* = 0.01 and 0.023 resp.). IL-6 correlated positivity with the presence of infection and type of pathogen (*P* = 0.038 and 0.034 resp.). Gram-negative bacteria were associated with higher significant IL-6 in comparison between others (196.4 ± 239.1 pg/dL; *P* = 0.011) but insignificantly affected NIV outcome (*P* > 0.05). *Conclusions*. High systemic inflammation could predict failure of NIV. G-ve bacteria correlated with high IL-6 but did not affect the response to NIV.

## 1. Introduction

Chronic obstructive pulmonary disease (COPD) is a chronic, progressive disease classified as a first class health problem. It is the 4th leading cause of death for adults in the United States [1] and the 6th in Egypt [2]. It is now well-established, indeed a defining characteristic [1], that COPD is associated with airway inflammation which is fundamental to its pathogenesis [3], and numerous individual studies have demonstrated the presence of systemic inflammation in stable COPD [4]. Acute exacerbations of chronic obstructive pulmonary disease (AECOPD) describe the phenomenon of sudden worsening in airway function and respiratory symptoms in patients with COPD [5]. Acute respiratory failure (ARF) is a common and important event, which is frequently associated with severe exacerbations of COPD and considered to be as decompensated COPD [6].

Bacterial infections are implicated in the majority of AECOPD episodes. Sputum and bronchoscopy data have shown that *Moraxella catarrhalis*, *Haemophilus influenzae*, and *Streptococcus pneumoniae* are the most common organisms associated with AECOPD episodes. Other bacteria (e.g., *Pseudomonas* and *Staphylococcus*) have also been implicated [7]. Atypical microorganisms such as *Mycoplasma pneumoniae* and *Chlamydia pneumoniae* have been implicated in 5–10% of AECOPD cases [8].

Recently, there is abundant evidence that exacerbations also are associated with increases in both pan-airway and systemic inflammatory markers [9]. It is reasonable to assume that worsening airway inflammation is the primary inciting event of AECOPD that may be caused by bacteria, viruses, or environmental pollutants, including cigarette smoke [10]. Abundant markers reported to be higher in blood during exacerbation compared with the baseline [11]. Yet there is no clear information about how these biomarkers relate to significant clinical outcomes such as length of hospital stay, need for ICU treatment, response to treatment, and mortality [12].

The aim of the present work was to study the value of some inflammatory biomarkers (Interleukin 6 (IL-6), interleukin 8 (IL-8), and C-reactive protein (CRP)) in predicting the outcome of noninvasive ventilation (NIV) as a therapeutic modality in the management of ARF on top of COPD. In addition, it aims to find out a possible relationship between these inflammatory markers' levels on one hand and arterial blood gases derangement, the presence of infection, the type of infection, and the bacteriological load in such patients on the other hand. 

## 2. Methods

### 2.1. Study Patients and Plan

The study included 33 patients attending the Respiratory Intensive Care Unit of the Chest Diseases Department, Alexandria Main University Hospital, Alexandria, Egypt. Patients included in this analysis were COPD patients as defined by the GOLD [1] without other significant respiratory diseases including asthma, tuberculosis, and bronchiectasis. All patients during enrollment in the study were in acute respiratory failure as defined by arterial blood gas criteria (PaO_2_ < 60 mmHg, with or without PaCO_2_ > 45 mmHg/pH < 7.35) during breathing room air [13]. However, any patient suffering from other confounding inflammatory diseases, such as malignancy, arthritis, connective tissue disorders, or inflammatory bowel disease, was excluded. 

All the patients on admission were subjected to thorough history taking which included name, age, sex, smoking index (pack/year), exacerbation history, drug history, and symptomatology including the assessment of dyspnea using “The Modified Medical Research Council (MMRC) dyspnea scale scoring” [14], full clinical examination, recording of the vital signs, the body mass index (BMI), some laboratory investigations (including complete blood picture, serum albumin, serum electrolytes, creatinine, and blood urea nitrogen). Furthermore, plain chest X-ray and arterial blood gases (ABG) were performed on admission. Sputum samples or bronchial wash using fibreoptic bronchoscopy were obtained on admission for microbiological analysis.

After the initial evaluation of the studied group of patients, they were managed according to the international guidelines. The patients were assigned to the standard drug protocol, supplemental oxygen therapy plus NIV. NIV was delivered for all studied cohort and maintained as long as it is tolerated. The administered FiO_2_ was adjusted to maintain oxygen saturation values of 88–92%. In the presence of any contraindications to NIV, which are (1) respiratory or cardiac arrest, (2) medical instability (hypotensive shock, myocardial infarction requiring intervention, or uncontrolled ischemia or arrhythmias), (3) unable to protect airway, (4) unable to fit mask, and (5) uncooperative or agitated, the patient was excluded from the study. The type of NIV was bilevel positive airway pressure (BIPAP) in most cases and only a minority of the cases benefited from continuous positive airway pressure (CPAP). The starting data applied for all cases was inspiratory positive airway pressure of 12 cm H_2_O and increased slowly as tolerated by the patients with a maximum of 25 cm H_2_O, while the expiratory positive airway pressure was 3 to 6 cm H_2_O. In case of CPAP, the pressure applied was 10 to 12 cm H_2_O. Failure of NIV was defined as termination of NIV trial and initiation of invasive mechanical ventilation. 

### 2.2. Microbiological Study

An equal volume of the sputum was mixed with sterile saline and incubated at room temperature for 15 minutes with intermittent shaking for homogenization of sputum. For the bronchial wash, no dilution was done. A semiquantitative method was used as follows.

- The homogenized sputum was diluted 1 to 100 in sterile saline (by adding 10 *μ*L from homogenized sputum to 990 *μ*L sterile saline).
- 10 *μ*L of the sample was inoculated per plate.
- The inoculum was spread confluently over one half of the plate and streaked over the other half.
- Plates were incubated 24 hours at 37°C. Plates inoculated included blood agar and MacConkey's agar.
- Assessing the bacteriological load was carried out by counting the growth of pathogens on the plates and correlating the numbers to the volume of the sample. A count of ≥10^6^ for sputum samples or ≥10^4^ for bronchial lavage samples was considered significant.
- Identification of the infecting pathogens was carried out according to standard microbiological procedures including Gram staining and biochemical reactions.
- Antibiotic susceptibility testing was carried out on the isolated pathogens according to CLSI guidelines.

Polymerase Chain Reaction (PCR) was done on the collected sputum or bronchial lavage sample for detection of *Mycoplasmapneumoniae* and *Chlamydia pneumonia* as follows.

### 2.3. DNA Extraction

DNA was extracted from all samples using the GeneJET Genomic DNA Purification Kit (Fermentas, Thermo Scientific). 

Briefly, samples were digested with Proteinase K in the supplied Lysis Solution. RNA was removed by treating the samples with RNAase. The lysate was then mixed with ethanol and loaded on the purification column where the DNA binds to the silica membrane. Impurities were effectively removed by washing the column with the prepared wash buffers. Genomic DNA was then eluted under low ionic strength conditions with the Elution Buffer.

**(1) Amplification of Mycoplasma pneumoniae DNA Sequence (see [15])**

PCR amplifications were carried out using 5 *μ*L sample extracts in a total volume of 25 *μ*L. Reactions were carried out using the ready-made master mix DreamTaq DNA Polymerase (Fermentas, Thermo Scientific) supplied in 2X DreamTaq Green Buffer (12.5 *μ*L) containing 0.4 mM each of dATP, dCTP, dGtp, and dTTP and 4 mM MgCl_2_. Twenty-five picomoles of each of the primers were added, then the volume was brought up to 25 *μ*L using nuclease free water. Positive (Mycoplasma DNA) and negative controls (water) were run concurrently with each run.

The two oligonucleotide primers flank a region 280 base pairs (bp) of the genome and have a sequence of 5′ GGG AGC AAA CAG GAT TAG ATA CCC T 3′ 5′ TGC ACC ATC TGT CAC TCT GTT AAC CTC 3′.

After an initial denaturation step at 94°C for 3 min, forty cycles were carried out under the following conditions: denaturation at 94°C for 45 sec, annealing at 55°C for 1 min, and extension at 72°C for 2 min. An additional step of extension at 72°C for 10 min was performed at the end of the 40 cycles. A second amplification of the PCR products was done using 5 *μ*L of the first amplification products as a PCR target and proceeding with the same PCR conditions. Because of high sensitivity of the PCR reaction used, stringent precautions were taken to avoid the risk of false-positive results.

Amplifications were carried out in a Techne Progene thermal cycler.

**(2) Amplification of Chlamydia pneumoniae DNA Sequence (see [16])**

PCR amplifications were carried out using 5 *μ*L sample extracts in a total volume of 25 *μ*L. Reactions were carried out using the ready-made master mix DreamTaq DNA Polymerase (Fermentas, Thermo Scientific) supplied in 2X DreamTaq Green Buffer (12.5 *μ*L) containing 0.4 mM each of dATP, dCTP, dGTP, and dTTP and 4 mM MgCl_2_. Fifteen picomoles of each of the primers were added, and the reaction volume was brought up to 25 *μ*L using nuclease-free water. Positive (Chlamydia DNA) and negative controls (water) were run concurrently with each run.

The two oligonucleotide primers flanking a region 474 base pairs (bp) of the *Chlamydia pneumoniae* gene had a sequence of HM-1: 5′ GTG TCA TTC GCC AAG GTT AA 3′ HR-1: 5′ TGC ATA ACC TAC GGT GTG GTT 3′.

Amplifications were carried out in a Techne Progene thermal cycler. After an initial denaturation step at 95°C for 5 min, forty cycles were carried out under the following conditions: denaturation at 94°C for 1 min, annealing at 48°C for 1 min, and extension at 72°C for 1 min. An additional step of extension at 72°C for 10 min was performed at the end of the 40 cycles. A second amplification of the PCR products was done using 5 *μ*L of the first amplification products as a PCR target and proceeding with the same PCR conditions. Because of high sensitivity of the PCR reaction used, stringent precautions were taken to avoid the risk of false-positive results.

**(3) Detection of the Amplified Products (see [15])**

Ten *μ*L of the reaction mixture were electrophoresed in 1% agarose gel in 0.09 M Tris-borate, pH 8.0, and 2 mM Na_2_EDTA (TBE) buffer with 100 bp molecular weight markers (Promega). Electrophoresis was carried out at 80 volts for 25 minutes. DNA fragments (280 bp for *Mycoplasma pneumoniae* and 474 bp for *Chlamydia pneumoniae* were visualized by ethidium bromide staining against an ultraviolet transilluminator.

### 2.4. Serum Samples and Analysis of the Inflammatory Markers

Serum samples were obtained on admission were preserved in −80°C till the end of the study period. The collected samples were analyzed for IL-6, IL-8, and CRP. The IL-6 and IL-8 were measured in serum samples by ELISA kit (AviBion human IL-6 and IL-8 and code number IL06001 and IL08001, respectively, Finland) according to the manufacturers' directions.

- An IL-6/IL-8 monoclonal coating antibody was adsorbed onto microwells.
- IL-6/IL-8 present in the sample or standard bound to antibodies adsorbed to the microwells; a biotin conjugated polyclonal IL-6/IL-8 antibody was added and bound to IL-6/IL-8 captured by the first antibody.
- Following incubation, unbound biotin conjugate was removed during a wash step. HRP-Streptavidin solution was added and binds to the biotin conjugated anti-IL-6/anti-IL-8. HRP-Streptavidin solution was removed during a wash step, and TMB One-Step Substrate reagent was added to each well.
- A colored product is formed in proportion to the amount of IL-6/IL-8 present in the sample. The reaction was terminated by addition of acid and absorbance (stop solution “2 N H2SO4”). The test was measured at 450 nm within 15 minutes. A standard curve was prepared from the eight IL-6/IL-8 standard dilutions. The standard curve points were 500, 250, 125, 62.5, 31.25, 15.62, and 0 pg/mL. Both IL-6/IL-8 sample concentrations were determined by plotting against the standard curve.

CRP was measured in serum samples by CRP-ultrasensitive (MICRO CRP/ULTRA CRP, Vital Diagnostics, Italy). This kit utilizes quantitative turbidimetric latex technique.

### 2.5. Statistical Analysis

Statistical analysis was carried out using Microsoft Excel and Statistical Package for Social Sciences (SPSS version 11, Chicago, IL, USA). Data are presented as means ± standard deviation (SD) and range. Categorical variables were described using frequencies and percentages. All statistical tests, unless otherwise stated, were employed. Kruskal-Wallis' test was used to compare the three independent groups for abnormally distributed data; Mann-Whitney's test was used to compare two independent groups for abnormally distributed data; the Monte-Carlo's test was used for testing associations; Spearman's rank correlations were used as well. The use of the inflammatory biomarkers (IL-6, CRP, and IL-8) in assessing the response to NIV was evaluated using a receiver operating characteristic (ROC) curve analysis. The area under the ROC curve (AUC) is a measure of how well each of IL-6, CRP, and IL-8 can distinguish between the two groups. In an ROC curve, the true positive rate (sensitivity) is plotted in function of the false-positive rate (100-specificity) for different cutoff points of each of IL-6, CRP, and IL-8. Each point on the ROC curve represents a sensitivity/specificity pair corresponding to a particular decision threshold. A test with perfect discrimination (no overlap in the two distributions) has an ROC curve that passes through the upper left corner (100% sensitivity, 100% specificity). Therefore, the closer the ROC curve is to the upper left corner, the higher the overall accuracy of the test. Statistical significance was accepted as *P* ≤ 0.05. All applied statistical tests of significance were two sided. 

## 3. Results

### 3.1. Baseline Patient Characteristics

Baseline clinical characteristics of the 33 patients included in the present study are reported in Table 1.

According to the response to the NIV, the patients were classified into responders (25 patients (75.8%)) and non-responders (8 patients (24.2%)). From the responder group, four patients died (12%). Equally, four patients (12%) died from the non-responder group. The comparison between both groups on admission was demonstrated in Table 2. There was a statistically significant difference between the responder and non-responder groups regarding dyspnea grade (2.4 ± 0.87 (range = 1–4) versus 3.4 ± 0.92 (range = 2–4)) (*P* = 0.023). However, there was no significant difference between the two groups regarding the age, BMI, smoking index, smoking status, the infection with Gram-negative bacteria, laboratory investigations, and FEV_1_ percentage predicted (*P* > 0.05). Furthermore, there was neither statistical difference between the remaining laboratory investigations nor between the ABG parameters, the vital signs.

#### 3.1.1. The Microbiological Analysis

**Figure 1:**
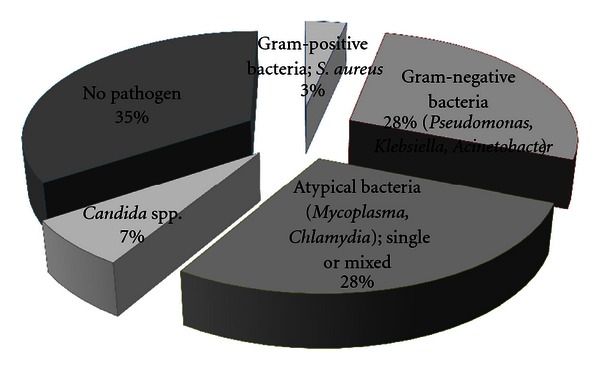
The bacteriological profile of the studied cases.

A wide spectrum of bacteria was revealed from examination of the sputum samples (Figure 1). From the studied cases, 4 patients (12% of the total number of studied patients “33”) were not able to give up sputum even after induction. Bronchoscopic lavage was performed for 4 patients (12%): 3 patients (9%) were on invasive mechanical ventilation after early failure of NIV and the one patient (3%) was from the responder group of NIV. A Gram-positive bacterium, *Staphylococcus aureus, *was detected in one sputum sample (3%). Gram-negative bacteria were detected in 8 sputum samples (28%): they were *Klebsiella* species in 3 (10%), *Pseudomonas* in 2 (7%), and *Acinetobacter* in 3 (10%). Fungal infection, that is, *Candida *species, was detected in 2 (7%).

Atypical pathogens were positive, using PCR amplification of the sputum samples, in 8 patients (28%). On one hand, five samples (17%) showed single atypical organism infection either *Mycoplasma pneumoniae* (in 4 samples (14%)) or *Chlamydia pneumoniae* (in one (3%)). On the other hand, mixed infections were detected in 3 samples (10%), that is, *M. pneumoniae* with others (Gram-negative bacterium in one sample or Gram-positive bacterium in 2 samples). However, no pathogen was detected in 35% of the studied samples. 

The Gram-negative bacteria were more prevalent among the non-responder group (57%) versus 22.7% among the responder group, and no evidence of infection was more frequent among the responder group (36.4%) versus 28.6% among non-responder group. However, the type of bacteria did not affect the response to NIV (*P* = 0.148).

#### 3.1.2. The Serum Inflammatory Markers

A comparison between the responder and non-responder groups is demonstrated in Table 3 regarding the biomarkers. The mean value of the IL-8 on admission was 43.7 pg/dL (±79.2) versus 171.9 pg/dL (±134.3) among the responders and non-responders, respectively. There was a statistically significant difference when comparing both groups (*P* = 0.01). No similar significance was reported regarding the IL-6 or CRP when comparing both groups.

**Figure 2:**
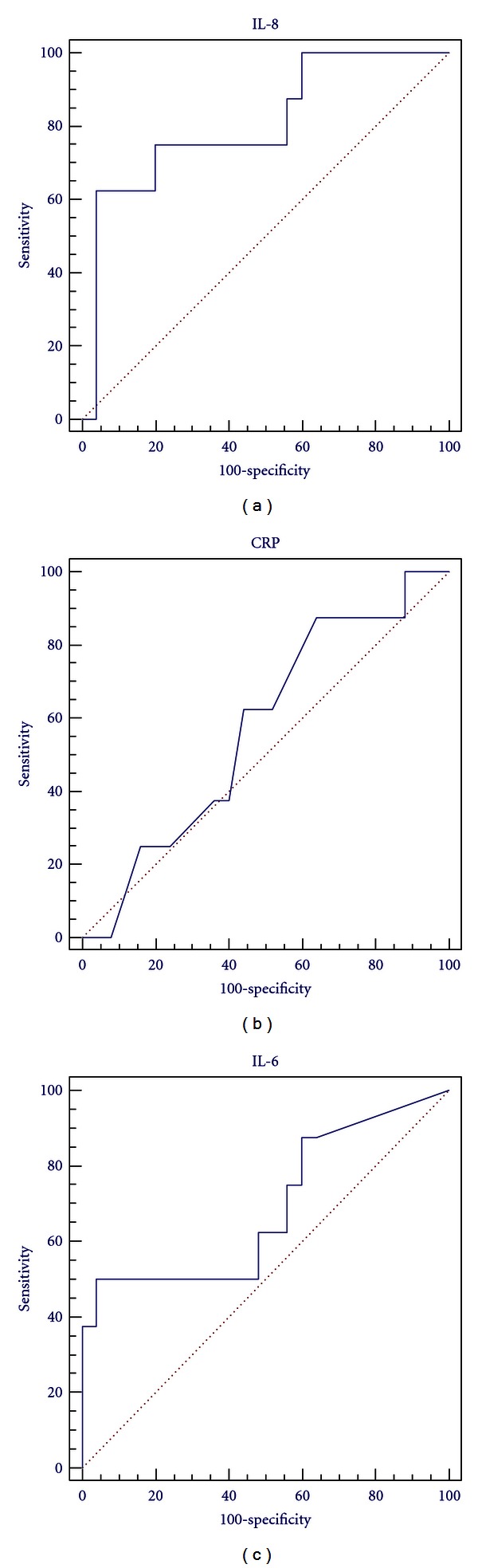
Receiver operating characteristic (ROC) curve for IL-6 on admission for the non-response to NIV (AUC = 0.688), ROC curve for IL-8 (AUC = 0.801), and ROC curve for CRP (AUC = 0.573).

With further analysis by the ROC (Figure 2), the best performing biomarker using the AUC was the IL-8 (AUC = 0.801). The best cutoff point for IL-8 regarding the “non-response” to NIV was >29.5 pg/dL; it has a sensitivity of 75% and a specificity of 80%. While that for IL-6 was >75.8 pg/dL with sensitivity of 50% and a specificity of 96% (AUC = 0.688). Regarding the CRP, the cutoff point was >2.95 mg/L, with a sensitivity of 62.5% and a specificity of 56% (AUC = 0.573). Accordingly, the cutoff point for both IL-6 and CRP had a low sensitivity which is prohibiting their use for prediction of NIV failure.

### 3.2. Correlations

**Figure 3:**
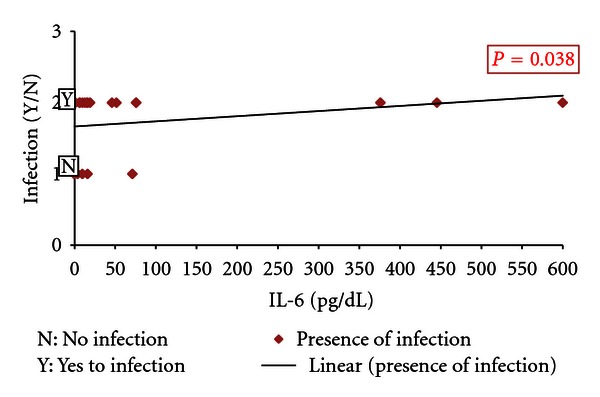
Correlation between the level of IL-6 on admission and the presence of infection.

**Figure 4:**
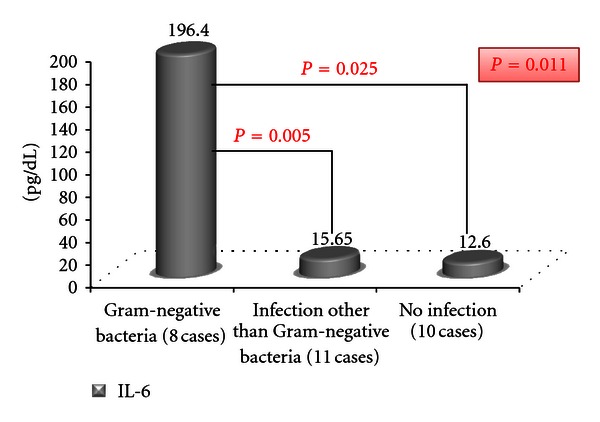
Comparison between the levels of IL-6 among the cases in relation to type of infection.

On admission, there was a statistically positive significant correlation between the IL-6 and the presence of infection regardless of its type (*P* = 0.038) (Figure 3). Furthermore, a statistically significant association between the level of IL-6 and the type of infection was present (*P* = 0.034) (Table 4). However, neither IL-8 nor CRP had a statistically significant difference when correlating either the presence of infection or the type of infection (*P* > 0.05). On classification of the pathogens into three groups, there was a significant difference between the readings of the IL-6 among the patients who were infected with Gram-negative bacteria and those who had no infection as well as those who were infected by pathogens other than Gram-negative bacteria (Figure 4). 

Nonetheless, there was no significant correlation between the studied biomarkers on admission and dyspnea scale, BMI, FEV_1_% predicted, ABG parameters, the comorbidities or bacteriological load (*P* > 0.05). 

## 4. Discussion

ARF on top of AECOPD represent a life-threatening condition. NIV should be used whenever possible as it has been shown to be an effective treatment for respiratory failure during AECOPD [17]. According to the present results, 75.8% succeeded the trial of NIV while 24.2% failed. Nearby results were recorded by Putinati et al. [18], as they reported a success among 77% and failureamong 23% of their patients episodes of respiratory failure. Also, Aburto et al. [19] had 75.3% of their patients received NIV successfully and 11.6% failed the NIV and were subjected for invasive mechanical ventilation. Other older studies also reported a range of failure of NIV between 7% and 24% [20–22].

The causes of failure of NIV among the non-responder group were mainly incoordination and deterioration of the consciousness. Ambrosino et al. [23] and Carlucci et al. [24] found that poor clinical tolerance of NIV was highly predictive of NIV failure. Soo Hoo et. al*.,* [25] observed that patients successfully treated with NIV were able to tolerate the mask longer than patients who failed NPPV. Other causes associated with failure of NIV in the present study were worsening of the ABG and starting hemodynamic instability which was unresponsiveness to fluid therapy.

Neither the ABG parameters nor the vital signs had a statistically significant difference between the responder and non-responder groups (*P* > 0.05). Accordingly, these parameters failed to predict the failure or success of NIV in the present work which were of significance in other studies [20, 23, 24, 26, 27].

The dyspnea grade as measured by MMRC was significantly higher in the non-responder group than the responder group. None of the other compared parameters showed any significance when comparing both groups. Moretti et al. [28] found no significant difference between the failure and success groups regarding the albumin, the electrolytes, the pulmonary function, and the presence of community acquired pneumonia. However, there was a statistically significant difference between the groups regarding the presence of comorbidities and “activity of daily living score” on admission which reflects the clinical severity of the disease. A recent report found that dyspnea may better reflect the complex functional and psychological impact of the disease rather than the lung function measurements [29]. Accordingly, high dyspnea grade indicates more severe clinical condition which could precipitate NIV failure in the present study.

The percentage of Gram-negative infection was higher among the non-responder group than the responder one (57% versus 22.7%) despite being statistically insignificant. Ferrer et al. [30] found that airway colonization by nonfermenting Gram-negative bacilli is strongly associated with NIV failure. The insignificant difference in the current study could be attributed to the small number of the non-responder group in comparison to the responder group.

The overall hospital mortality was 24% in the present study, which is within the range of values reported by Baldwin and Allen [31] for NIV (6–25%). Furthermore, the hospital mortality rates were similar in both groups (responders and non-responders; each 12%) which was similar to that reported by Conti et al. [32] and Squadrone et al. [33]. Worthwhile of noting, NIV is not a therapy, but it is a form of life support until the cause underlying the acute respiratory failure is reversed with medical therapy [34].

Interestingly, in the present study, there was a statistically significant difference between the responder and non-responder groups regarding the IL-8 with higher levels among the non-responders. However, neither CRP nor IL-6 had similar results when comparing both groups. In the literature, inflammation at AECOPD becomes more marked with recruitment of neutrophils and eosinophils, the major components of the inflammatory response [35, 36], and increased CD4^+^ lymphocytes in the bronchial mucosa [37, 38]. This is associated with increased markers of airway neutrophilic inflammation (myeloperoxidase, IL-8, IL-6, and TNF-*α*) at the time of acute exacerbation [39, 40]. In contrast to stable disease, exacerbations appear to be associated with a direct correlation between the degree of airway inflammation and the magnitude of the systemic acute-phase response which supports the hypothesis of “spilling over” [9].

IL-8 is a potent neutrophil chemokine and activator, which can induce the migration of neutrophils to the airway and promote neutrophils' degranulation [40]. Qiu et al.*'*s [41] study of patients with COPD exacerbations has shown an upregulation of gene expression for IL-8 and epithelial-derived neutrophil attractant-78 in intubated patients with severe exacerbations. Elevated serum IL-8 in the present study could be a mirror of severe airway inflammation in severe AECOPD irrespective to the cause of the ARF. The best working cutoff point for IL-8 was 29.5 pg/dL as above this value the sensitivity for non-responding to NIV trial is supposed to be 75% with acceptable specificity (80%). Serum IL-6, CRP, and the total WBC were higher among the non-responder group despite being insignificant. Bathoorn et al. [42] reported increased systemic inflammation as demonstrated by increased number of blood total leukocytes, IL-6, and a trend in CRP during exacerbations. Accordingly, high systemic inflammation was associated with NIV failure which in turn reflects accentuated airway inflammation.

According to previously published data, about 50–70% of exacerbations are due to respiratory infections [43]. Commonly isolated organisms include *H. influenzae*, *S. pneumoniae*, *M. catarrhalis, H. parainfluenzae *and* P. aeruginosa*, with other Gram-negative bacteria occurring more rarely [44]. In the present study, the bacteriological profile showed a wide spectrum of infective pathogens. Respiratory infections were the cause of exacerbation in 66% of the studied samples. The Gram-negative bacteria constituted the most common causes of AECOPD either single or mixed (31% of infection). Similarly, Miravitlles et al. [45], Eller et al. [46], and Bogaert et al. [47], utilizing sputum cultures, demonstrated an increasing frequency of isolation of *Pseudomonas *spp and other Gram-negative bacilli in AECOPD. Soler et al. [48] also found a high incidence of *Pseudomonas aeruginosa and* other Gram-negative bacilli (28%) among their patients presenting with AECOPD. Accordingly, infections with *Pseudomonas *spp and Gram-negative bacteria occur in more severe exacerbations mostly, affecting the most debilitated patients. Another recent study by Li et al. [49] found that only 47% of theCOPD-exacerbationpatients had a positive sputum culture. They reported Gram-negative bacteria to be the most prevalent in their cohort. The high incidence of Gram-negatives could be related to antibiotic selection pressure, exposure to hospital flora, or the degree of host immune compromise [50].

Another observation in the present work was the incidence of atypical infection which was higher than the literature (28% either single or copathogen). Moreover, the *Mycoplasma pneumonia *was more frequent than *Chlamydia pneumonia *(25% versus 3%).

Meloni et al. [8] found that *C. pneumoniae* infection was reported in 8.9% of AECOPD patients and acute *M. pneumoniae* infection was found in 6.7% of the AECOPD. Lieberman et al. [51] and Papaetis et al. [52] found *M. pneumoniae* to be the cause of AECOPD in 14% and 9%, respectively. Otherwise, *C. pneumoniae* has been reported to cause 4–16% of AECOPD [53, 54]. The difference between these results and ours is because former studies were based almost solely on serological evidence, while the present study relied on detection of the organisms in sputum samples using PCR technique. PCR is considered to be the preferred diagnostic procedure for the diagnosis of *M. pneumoniae* infections [55]. Additionally, the high atypical bacterial infection in the studied cohort could reflect the high prevalence of this bacterial group in the community.

It was reported that the prevalence of atypical bacteria in Africa as a cause of lower respiratory tract infection is 20% [56]. Lui et al. [57] also found that atypical bacteria constituted 28.6% of the causal organisms of CAP where *M. pneumonia *and *C. pneumoniae*, as single or copathogens, were the commonest. Seemungal et al. [58] and Blasi et al. [59] found that 28% and 38%, respectively, of their patients had *C. pneumoniae* DNA PCR positive in their sputum during the AECOPD. But both Diederen et al. [60] and Varma-Basil et al. [61] found that all samples collected from AECOPD were negative for *M. pneumoniae* and *C. pneumoniae* DNA. The difference in the results between the present study and others may be due to a number of reasons, including differences in PCR techniques used [62, 63] and the differences in study plan and subjects. 

There was a statistically significant correlation between the serum IL-6 and the type of bacteria. Furthermore, the positive significance was related to presence of Gram-negative bacteria rather than other types. Accordingly, the Gram-negative bacteria were associated with higher systemic inflammation. This could be explained on the basis of the endotoxins especially the lipopolysaccharides (LPS) which constitute the major cell wall component in all Gram-negative bacteria [64]. LPS initiates downstream intracellular signaling pathways that ultimately result in the activation of the nuclear transcription factor, nuclear factor *κ*B [65], which in turn stimulates the transcription of genes coding for proinflammatory cytokines such as tumor necrosis factor-*α* and interleukin-1*β* which appear early in the circulation followed by IL-6 appearance within 2 hours [66]. Khair et al. [67] found that cultures of bronchial epithelial cells showed increased IL-6 production in response to *Haemophilus influenzae* endotoxin. Hence, increased serum IL-6 in accordance with Gram-negative infection is not surprising due to associated endotoxin.

## 5. Conclusions

Neither ABG nor vital signs were predictors of NIV failure. High serum IL-8 as a marker of systemic inflammation and poor baseline functional state (expressed by higher dyspnea grade) could predict failure of NIV. The presence of infection accentuates the systemic inflammation accompanied the ARF on top of AECOPD which was expressed by elevation of IL-6. Atypical and Gram-negative bacteria constitute the most common pathogens of AECOPD. Moreover, Gram-negative bacteria correlated with high IL-6 but did not affect the response to NIV. 

## Figures and Tables

**Table 1:** Baseline clinical characteristics of the patients.

Baseline characteristics	Cases	Percentage (%)
Age (years)		
Mean ± SD (range)	56.61 ± 7.87 (45–81)	
Gender		
Male/female	26/7	(79%)/(21%)
Smoking status		
Current smokers	11	(33%)
Ex-smokers	15	(46%)
Passive smokers	7	(21%)
Smoking index (p/yr)		
Mean ± SD (range)	40.25 ± 23 (10–120)	
BMI (kg/m2)		
Mean ± SD (Range)	28.8 ± 8 (12.7–42)	
Presence of comorbidities	18	(54%)
Hypertension	13	(37%)
Diabetes mellitus	7	(21%)
Others	6	(17%)
Ischemic heart disease	3	(9.1%)
History of deep vein thrombosis	2	(6.1%)
Liver cirrhosis	1	(3%)
Sleep apnea syndrome (OSA)	10	(30.3%)
History of exacerbation		
Less than or 3 times/year	11	(33%)
Previous mechanical ventilation	3	(9%)
More than 3 times/year	22	(67%)
Previous mechanical ventilation	6	(18%)

OSA: obstructive sleep apnea, p/yr: pack/year.

**Table 2:** Comparison between different parameters among the responder and non-responder groups measured on admission.

	Responders (*N* = 25 cases)	Nonresponders (*N* = 8 cases)	Significance (*P*)
Age (yrs)	56.64 ± 8.18 (45–81)	56.5 ± 7.29 (45–65)	0.359 (0.719)
BMI (kg/m^2^)	27.7 ± 7.62 (16.6–44.1)	28 ± 9.2 (12.7–40.8)	0.231 (0.817)
Dyspnea (MMRC)	2.4 ± 0.87 (1–4)	3.4 ± 0.92 (2–4)	**2.277 ** ** (0.023)** ^ ∗^
Current smoker (*N* (%))	8 (32%)	2 (25%)	^FE^ *P* = 1.0
Infection with Gram-negative, yes/no (%)	5/22 (22.7%)	4/7 (57%)	^FE^ *P* = 0.158
FEV_1_ percentage predicted	36.7 ± 17.1% (20.1–99.4)	35.1% (22.8–48.5)	1.638 (0.101)
Laboratory investigations			
Total WBC (10^3^/*μ*L)	11.3 ± 7.5 (4.1–37.5)	13.4 ± 5.4 (7.44–21.51)	1.47 (0.141)
Na (mmol/L)	138.3 ± 3.7 (131–146)	134.8 ± 10.5 (114–151)	1.182 (0.237)
K (mmol/L)	4.4 ± 0.6 (3.5–5.8)	4.1 ± 0.6 (3.5–5.1)	0.822 (0.411)
BUN (mg/dL)	25.4 ± 15.9 (9–73)	34.1 ± 13.9 (13–54)	1.704 (0.088)
Cr (mg/dL)	0.9 ± 0.5 (0.4–3)	1.4 ± 0.7 (0.6–2.6)	1.904 (0.057)
Serum albumin (g/dL)	3.0 ± 0.5 (2.1–4)	2.7 ± 0.3 (2.4–3.2)	1.682 (0.093)
ABG			
pH	7.31 ± 0.06 (7.21–7.45)	7.34 ± 0.08 (7.23–7.451)	0.742 (0.458)
PCO_2_ (mmHg)	67.2 ± 16 (38.6–106)	68.3 ± 27.8 (23.6–103)	0.653 (0.514)
PO_2_ (mmHg)	48. 8 ± 14.2 (25–80)	42.4 ± 4.4 (35.1–48)	1.088 (0.277)
Vital signs			
HR	100 ± 11	103 ± 26	−0.167 (0.865)
SBP	141 ± 27	135 ± 22	−0.466 (0.641)
RR	29 ± 4	32 ± 7	−1.078 (0.281)

Yrs: years, MMRC: the Modified Medical Research Council, FEV_1_: forced expiratory volume in 1 second, WBC: white blood count, Na: sodium, K: potassium, BUN: blood urea nitrogen, Cr: creatinine, ABG: arterial blood gases, SBP: systolic blood pressure, RR: respiratory rate, HR: heart rate, pk/yr: pack/year, PaO_2_ (mmHg): partial arterial pressure of oxygen, PaCO_2_ (mmHg): partial arterial pressure of carbon dioxide, *N*: number of cases,^∗^significant if *P* ≤ 0.05, ^FE^ *P* = Fisher's exact test.

**Table 3:** Comparison between the levels of studied biomarkers according to the response to NIV.

Biomarkers	Response to NIV		Significance (*P*)
	Responder	Non-responder
IL-6 (pg/dL)	31.5 ± 75.6	214.4 ± 275.1	*z* = 1.59 (0.11)
IL-8 (pg/dL)	43.7 ± 79.2	171.9 ± 134.3	**z =** **2.563 (0.01)** ^∗^
CRP (mg/L)	2.8 ± 1.1	2.9 ± 0.9	*z* = 0.611 (0.541)

IL-6: interleukin 6, IL-8: interleukin 8, CRP: C-reactive protein, NIV: noninvasive ventilation, ^∗^significant at *P* ≤ 0.05 between the responders and nonresponders.

**Table 4:** Comparison between the levels of the studied biomarkers and the type of infection on admission.

Biomarker			Infection				Significance
	Gram-negative bacteria (8 cases)	Gram-positive bacteria (1 case)	Fungal (2 cases)	Atypical infection (5 cases)	Mixed (*mycoplasma* and others) (3 cases)	No infection (10 cases)
IL-6 (pg/dL)	196.4 ± 239.1	12.8	35.4 ± 22.91	17.72 ± 32.57	5.2 ± 5.2	10.67 ± 21.94	**χ** ^2^ = 12.03
							**P** = 0.034*
IL-8 (pg/dL)	96.96 ± 118.32	11.8	18.75 ± 14.78	39.1 ± 52.88	43.87 ± 43.87	87.53 ± 134.54	*χ* ^2^ = 0.839
							*P* = 0.975
CRP (mg/L)	2.84 ± 1.11	3.4	2.85 ± 0.78	3.04 ± 0.78	2.83 ± 2.83	2.55 ± 1.11	*χ* ^2^ = 1.54
							*P* = 0.908

## Abbreviations

COPD: Chronic obstructive pulmonary disease
AECOPD: Acute exacerbations of chronic obstructive pulmonary disease
ARF: Acute respiratory failure
NIV: Noninvasive ventilation
IL-6: Interleukin 6
IL-8: Interleukin 8
CRP: C-reactive protein
GOLD: The Global Initiative for Chronic Obstructive Lung Disease
MMRC: The Modified Medical Research Council
BMI: Body mass index
ABG: Arterial blood gases
FiO_2_: Fraction of inspired oxygen
BIPAP: Bilevel positive airway pressure
CPAP: Continuous positive airway pressure
PCR: Polymerase chain reaction
bp: base pairs
SD: Standard deviation
ROC: Receiver Operating characteristic curve
AUC: The area under the ROC curve
OSA: Obstructive sleep apnea
p/yr: Pack/year
FEV_1_: Forced expiratory volume in 1 second
yrs: years
WBC: White blood count
Na: Sodium
K: Potassium
BUN: Blood urea nitrogen
Cr: Creatinine
SBP: Systolic blood pressure
RR: Respiratory rate
HR: Heart rate
PaO_2_ (mmHg): Partial arterial pressure of oxygen
PaCO_2_ (mmHg): Partial arterial pressure of carbon dioxide
N: Number of cases
LPS: lipopolysaccharides.
